# Supplementary material for: Bioactivity Potential of Bioceramic-Based Root Canal Sealers: A Scoping Review
Source: Life (Basel). 2022 Nov 11;12(11):1853. doi: 10.3390/life12111853 (PMC9697500; doi:10.3390/life12111853)
Supplement: Supplementary file 1 [file life-12-01853-s001.zip › life-1968786-supplementary.pdf]

**Supplementary Table S1** – Search strategies	
	**Search Terms**
PubMed/MEDLINE	
#4	Search #1 AND #2 AND #3
#3	Search (Root Canal Filling Material) OR (Root Canal Sealants) OR (Sealants, Root Canal) OR (Canal Sealant, Root) OR (Canal Sealants, Root) OR (Root Canal Sealant) OR (Sealant, Root Canal)
#2	Search (Bioceramics) OR (Bioceramic) OR (Calcium silicate) OR (Tricalcium silicate) OR (Mineral trioxide aggregate) OR (Calcium phosphate) OR (Calcium silicate-based) OR (Tricalcium silicate-based) OR (Silicates)
#1	Search (Bioactive) OR (Bioactivity) OR (Bioactivated) OR (Bioactivates) OR (Bioactivation) OR (Bioactivities) OR (Products, Biological) OR (Biological Product) OR (Product, Biological) OR (Biologic Product) OR (Product, Biologic) OR (Biologic Products) OR (Biopharmaceuticals) OR (Biopharmaceutical) OR (Biological) OR (Biologic) OR (Biological Drug) OR (Drug, Biological) OR (Biologic Drugs) OR (Drugs, Biologic) OR (Biological Medicine) OR (Medicine, Biological) OR (Biological Medicines) OR (Medicines, Biological) OR (Biologicals) OR (Biologic Medicines) OR (Medicines, Biologic) OR (Biologic Pharmaceuticals) OR (Pharmaceuticals, Biologic) OR (Biologics) OR (Biological Factors) OR (Biologic Drug) OR (Drug, Biologic) OR (Biological Drugs) OR (Drugs, Biological) OR (Natural Products) OR (Natural Product) OR (Product, Natural) OR (Testing, Materials) OR (Testing, Biocompatible Materials) OR (Biocompatible Materials Testing) OR (Materials Testing, Biocompatible) OR (Biocompatibility Testing) OR (Testing, Biocompatibility) OR (Testings, Biocompatibility) OR (Hemocompatibility Testing) OR (Hemocompatibility Testings) OR (Testing, Hemocompatibility) OR (Testing, Hemocompatible Materials) OR (Hemocompatible Materials Testing) OR (Materials Testing, Hemocompatible) OR (Physiologic Calcification) OR (Calcification, Physiological) OR (Physiological Calcification) OR (Bone Mineralization) OR (Mineralization, Bone) OR (Mineralization) OR (Mineralisable) OR (Mineralized) OR (Bone Formation) OR (Ossification) OR (Ossifications) OR (Osteoclastogenesis) OR (Osteoclastogeneses) OR (Endochondral Ossification) OR (Endochondral Ossifications) OR (Ossification, Endochondral) OR (Ossifications, Endochondral) OR (Physiologic Ossification) OR (Ossification, Physiological) OR (Physiological Ossification) OR (Ossification, Physiologic) OR (angiogeneic) OR (angiogenically) OR (angiogenicity) OR (angiogenics) OR (angiogens) OR (Proliferation, Cell) OR (Cellular Proliferation) OR (Proliferation, Cellular) OR (Cell Multiplication) OR (Multiplication, Cell) OR (Cell Growth in Number) OR (Cell Number Growth) OR (Growth, Cell Number) OR (Number Growth, Cell) OR (gene expression) OR (odontogenic) OR (odontogenous) OR (osteogene) OR (osteogeneic)
Embase	
#4	Search #1 AND #2 AND #3
#3	Search (Root Canal Filling Material) OR (Root Canal Sealants) OR (Sealants, Root Canal) OR (Canal Sealant, Root) OR (Canal Sealants, Root) OR (Root Canal Sealant) OR (Sealant, Root Canal)
#2	Search (Bioceramics) OR (Bioceramic) OR (Calcium silicate) OR (Tricalcium silicate) OR (Mineral trioxide aggregate) OR (Calcium phosphate) OR (Calcium silicate-based) OR (Tricalcium silicate-based) OR (Silicates)
#1	Search (Bioactive) OR (Bioactivity) OR (Bioactivated) OR (Bioactivates) OR (Bioactivation) OR (Bioactivities) OR (Products, Biological) OR (Biological Product) OR (Product, Biological) OR (Biologic Product) OR (Product, Biologic) OR (Biologic Products) OR (Biopharmaceuticals) OR (Biopharmaceutical) OR (Biological) OR (Biologic) OR (Biological Drug) OR (Drug, Biological) OR (Biologic Drugs) OR (Drugs, Biologic) OR (Biological Medicine) OR (Medicine, Biological) OR (Biological Medicines) OR (Medicines, Biological) OR (Biologicals) OR (Biologic Medicines) OR (Medicines, Biologic) OR (Biologic Pharmaceuticals) OR (Pharmaceuticals, Biologic) OR (Biologics) OR (Biological Factors) OR (Biologic Drug) OR (Drug, Biologic) OR (Biological Drugs) OR (Drugs, Biological) OR (Natural Products) OR (Natural Product) OR (Product, Natural) OR (Testing, Materials) OR (Testing, Biocompatible Materials) OR (Biocompatible Materials Testing) OR (Materials Testing, Biocompatible) OR (Biocompatibility Testing) OR (Testing, Biocompatibility) OR (Testings, Biocompatibility) OR (Hemocompatibility Testing) OR (Hemocompatibility Testings) OR (Testing, Hemocompatibility) OR (Testing, Hemocompatible Materials) OR (Hemocompatible Materials Testing) OR (Materials Testing, Hemocompatible) OR (Physiologic Calcification) OR (Calcification, Physiological) OR (Physiological Calcification) OR (Bone Mineralization) OR (Mineralization, Bone) OR (Mineralization) OR (Mineralisable) OR (Mineralized) OR (Bone Formation) OR (Ossification) OR (Ossifications) OR (Osteoclastogenesis) OR (Osteoclastogeneses) OR (Endochondral Ossification) OR (Endochondral Ossifications) OR (Ossification, Endochondral) OR (Ossifications, Endochondral) OR (Physiologic Ossification) OR (Ossification, Physiological) OR (Physiological Ossification) OR

	(Ossification, Physiologic) OR (angiogeneic) OR (angiogenically) OR (angiogenicity) OR (angiogenics) OR (angiogens) OR (Proliferation, Cell) OR (Cellular Proliferation) OR (Proliferation, Cellular) OR (Cell Multiplication) OR (Multiplication, Cell) OR (Cell Growth in Number) OR (Cell Number Growth) OR (Growth, Cell Number) OR (Number Growth, Cell) OR (gene expression) OR (odontogenic) OR (odontogenous) OR (osteogene) OR (osteogeneic)
Web of Science	
#4	Search #1 AND #2 AND #3
#3	TS=((Root Canal Filling Material) OR (Root Canal Sealants) OR (Sealants, Root Canal) OR (Canal Sealant, Root) OR (Canal Sealants, Root) OR (Root Canal Sealant) OR (Sealant, Root Canal))
#2	TS=((Bioceramics) OR (Bioceramic) OR (Calcium silicate) OR (Tricalcium silicate) OR (Mineral trioxide aggregate) OR (Calcium phosphate) OR (Calcium silicate-based) OR (Tricalcium silicate-based) OR (Silicates))
#1	TS=((Bioactive) OR (Bioactivity) OR (Bioactivated) OR (Bioactivates) OR (Bioactivation) OR (Bioactivities) OR (Products, Biological) OR (Biological Product) OR (Product, Biological) OR (Biologic Product) OR (Product, Biologic) OR (Biologic Products) OR (Biopharmaceuticals) OR (Biopharmaceutical) OR (Biological) O (Biologic) OR (Biological Drug) OR (Drug, Biological) OR (Biologic Drugs) OR (Drugs, Biologic) OR (Biological Medicine) OR (Medicine, Biological) OR (Biological Medicines) OR (Medicines, Biological) OR (Biologicals) OR (Biologic Medicines) OR (Medicines, Biologic) OR (Biologic Pharmaceuticals) OR (Pharmaceuticals, Biologic) OR (Biologics) OR (Biological Factors) OR (Biologic Drug) OR (Drug, Biologic) OR (Biological Drugs) OR (Drugs, Biological) OR (Natural Products) OR (Natural Product) OR (Product, Natural) OR (Testing, Materials) OR (Testing, Biocompatible Materials) OR (Biocompatible Materials Testing) OR (Materials Testing, Biocompatible) OR (Biocompatibility Testing) OR (Testing, Biocompatibility) OR (Testings, Biocompatibility) OR (Hemocompatibility Testing) OR (Hemocompatibility Testings) OR (Testing, Hemocompatibility) OR (Testing, Hemocompatible Materials) OR (Hemocompatible Materials Testing) OR (Materials Testing, Hemocompatible) OR (Physiologic Calcification) OR (Calcification, Physiological) OR (Physiological Calcification) OR (Bone Mineralization) OR (Mineralization, Bone) OR (Mineralization) OR (Mineralisable) OR (Mineralized) OR (Bone Formation) OR (Ossification) OR (Ossifications) OR (Osteoclastogenesis) OR (Osteoclastogeneses) OR (Endochondral Ossification) OR (Endochondral Ossifications) OR (Ossification, Endochondral) OR (Ossifications, Endochondral) OR (Physiologic Ossification) OR (Ossification, Physiological) OR (Physiological Ossification) OR (Ossification, Physiologic) OR (angiogeneic) OR (angiogenically) OR (angiogenicity) OR (angiogenics) OR (angiogens) OR (Proliferation, Cell) OR (Cellular Proliferation) OR (Proliferation, Cellular) OR (Cell Multiplication) OR (Multiplication, Cell) OR (Cell Growth in Number) OR (Cell Number Growth) OR (Growth, Cell Number) OR (Number Growth, Cell) OR (gene expression) OR (odontogenic) OR (odontogenous) OR (osteogene) OR (osteogeneic))
SciVerse Scopus	
#3	Search #1 AND #2 AND #3
#2	TITLE-ABS-KEY (("Root Canal Filling Material") OR ("Root Canal Sealants") OR ("Sealants, Root Canal") OR ("Canal Sealant, Root") OR ("Canal Sealants, Root") OR ("Root Canal Sealant") OR ("Sealant, Root Canal"))
#1	TITLE-ABS-KEY (("Bioceramics") OR ("Bioceramic") OR ("Calcium silicate") OR ("Tricalcium silicate") OR ("Mineral trioxide aggregate") OR ("Calcium phosphate") OR ("Calcium silicate-based") OR ("Tricalcium silicate-based") OR ("Silicates"))
#1	TITLE-ABS-KEY (("Bioactive") OR ("Bioactivity") OR ("Bioactivated") OR ("Bioactivates") OR ("Bioactivation") OR ("Bioactivities") OR ("Products, Biological") OR ("Biological Product") OR ("Product, Biological") OR ("Biologic Product") OR ("Product, Biologic") OR ("Biologic Products") OR ("Biopharmaceuticals") OR ("Biopharmaceutical") OR ("Biological") OR ("Biologic") OR ("Biological Drug") OR ("Drug, Biological") OR ("Biologic Drugs") OR ("Drugs, Biologic") OR ("Biological Medicine") OR ("Medicine, Biological") OR ("Biological Medicines") OR ("Medicines, Biological") OR ("Biologicals") OR ("Biologic Medicines") OR ("Medicines, Biologic") OR ("Biologic Pharmaceuticals") OR ("Pharmaceuticals, Biologic") OR ("Biologics") OR ("Biological Factors") OR ("Biologic Drug") OR ("Drug, Biologic") OR ("Biological Drugs") OR ("Drugs, Biological") OR ("Natural Products") OR ("Natural Product") OR ("Product, Natural") OR ("Testing, Materials") OR ("Testing, Biocompatible Materials") OR ("Biocompatible Materials Testing") OR ("Materials Testing, Biocompatible") OR ("Biocompatibility Testing") OR ("Testing, Biocompatibility") OR ("Testings, Biocompatibility") OR ("Hemocompatibility Testing") OR ("Hemocompatibility Testings") OR ("Testing, Hemocompatibility") OR

	(“Testing, Hemocompatible Materials”) OR (“Hemocompatible Materials Testing”) OR (“Materials Testing, Hemocompatible”) OR (“Physiologic Calcification”) OR (“Calcification, Physiological”) OR (“Physiological Calcification”) OR (“Bone Mineralization”) OR (“Mineralization, Bone”) OR (“Mineralization”) OR (“Mineralisable”) OR (“Mineralized”) OR (“Bone Formation”) OR (“Ossification”) OR (“Ossifications”) OR (“Osteoclastogenesis”) OR (“Osteoclastogeneses”) OR (“Endochondral Ossification”) OR (“Endochondral Ossifications”) OR (“Ossification, Endochondral”) OR (“Ossifications, Endochondral”) OR (“Physiologic Ossification”) OR (“Ossification, Physiological”) OR (“Physiological Ossification”) OR (“Ossification, Physiologic”) OR (“angiogeneic”) OR (“angiogenically”) OR (“angiogenicity”) OR (“angiogenics”) OR (“angiogens”) OR (“Proliferation, Cell”) OR (“Cellular Proliferation”) OR (“Proliferation, Cellular”) OR (“Cell Multiplication”) OR (“Multiplication, Cell”) OR (“Cell Growth in Number”) OR (“Cell Number Growth”) OR (“Growth, Cell Number”) OR (“Number Growth, Cell”) OR (“gene expression”) OR (“odontogenic”) OR (“odontogenous”) OR (“osteogene”) OR (“osteogeneic”))
The Cochrane Library	
#4	Search #1 AND #2 AND #3
#3	TITLE-ABS-KEY (Root Canal Filling Material) OR (Root Canal Sealants) OR (Sealants, Root Canal) OR (Canal Sealant, Root) OR (Canal Sealants, Root) OR (Root Canal Sealant) OR (Sealant, Root Canal)
#2	TITLE-ABS-KEY (Bioceramics) OR (Bioceramic) OR (Calcium silicate) OR (Tricalcium silicate) OR (Mineral trioxide aggregate) OR (Calcium phosphate) OR (Calcium silicate-based) OR (Tricalcium silicate-based) OR (Silicates)
#1	TITLE-ABS-KEY (Bioactive) OR (Bioactivity) OR (Bioactivated) OR (Bioactivates) OR (Bioactivation) OR (Bioactivities) OR (Products, Biological) OR (Biological Product) OR (Product, Biological) OR (Biologic Product) OR (Product, Biologic) OR (Biologic Products) OR (Biopharmaceuticals) OR (Biopharmaceutical) OR (Biological) OR (Biologic) OR (Biological Drug) OR (Drug, Biological) OR (Biologic Drugs) OR (Drugs, Biologic) OR (Biological Medicine) OR (Medicine, Biological) OR (Biological Medicines) OR (Medicines, Biological) OR (Biologicals) OR (Biologic Medicines) OR (Medicines, Biologic) OR (Biologic Pharmaceuticals) OR (Pharmaceuticals, Biologic) OR (Biologics) OR (Biological Factors) OR (Biologic Drug) OR (Drug, Biologic) OR (Biological Drugs) OR (Drugs, Biological) OR (Natural Products) OR (Natural Product) OR (Product, Natural) OR (Testing, Materials) OR (Testing, Biocompatible Materials) OR (Biocompatible Materials Testing) OR (Materials Testing, Biocompatible) OR (Biocompatibility Testing) OR (Testing, Biocompatibility) OR (Testings, Biocompatibility) OR (Hemocompatibility Testing) OR (Hemocompatibility Testings) OR (Testing, Hemocompatibility) OR (Testing, Hemocompatible Materials) OR (Hemocompatible Materials Testing) OR (Materials Testing, Hemocompatible) OR (Physiologic Calcification) OR (Calcification, Physiological) OR (Physiological Calcification) OR (Bone Mineralization) OR (Mineralization, Bone) OR (Mineralization) OR (Mineralisable) OR (Mineralized) OR (Bone Formation) OR (Ossification) OR (Ossifications) OR (Osteoclastogenesis) OR (Osteoclastogeneses) OR (Endochondral Ossification) OR (Endochondral Ossifications) OR (Ossification, Endochondral) OR (Ossifications, Endochondral) OR (Physiologic Ossification) OR (Ossification, Physiological) OR (Physiological Ossification) OR (Ossification, Physiologic) OR (angiogeneic) OR (angiogenically) OR (angiogenicity) OR (angiogenics) OR (angiogens) OR (Proliferation, Cell) OR (Cellular Proliferation) OR (Proliferation, Cellular) OR (Cell Multiplication) OR (Multiplication, Cell) OR (Cell Growth in Number) OR (Cell Number Growth) OR (Growth, Cell Number) OR (Number Growth, Cell) OR (gene expression) OR (odontogenic) OR (odontogenous) OR (osteogene) OR (osteogeneic)
LILACS/BBO	
#4	Search #1 AND #2 AND #3
#3	Search (Root Canal Filling Material) OR (Root Canal Sealants) OR (Sealants, Root Canal) OR (Canal Sealant, Root) OR (Canal Sealants, Root) OR (Root Canal Sealant) OR (Sealant, Root Canal)
#2	Search (Bioceramics) OR (Bioceramic) OR (Calcium silicate) OR (Tricalcium silicate) OR (Mineral trioxide aggregate) OR (Calcium phosphate) OR (Calcium silicate-based) OR (Tricalcium silicate-based) OR (Silicates)
#1	Search (Bioactive) OR (Bioactivity) OR (Bioactivated) OR (Bioactivates) OR (Bioactivation) OR (Bioactivities) OR (Products, Biological) OR (Biological Product) OR (Product, Biological) OR (Biologic Product) OR (Product, Biologic) OR (Biologic Products) OR (Biopharmaceuticals) OR (Biopharmaceutical) OR (Biological) OR (Biologic) OR (Biological Drug) OR (Drug, Biological) OR (Biologic Drugs) OR (Drugs, Biologic) OR (Biological Medicine) OR (Medicine, Biological) OR (Biological Medicines) OR (Medicines, Biological) OR (Biologicals) OR (Biologic Medicines) OR (Medicines, Biologic) OR (Biologic Pharmaceuticals) OR (Pharmaceuticals, Biologic) OR (Biologics)

OR (Biological Factors) OR (Biologic Drug) OR (Drug, Biologic) OR (Biological Drugs) OR (Drugs, Biological) OR (Natural Products) OR (Natural Product) OR (Product, Natural) OR (Testing, Materials) OR (Testing, Biocompatible Materials) OR (Biocompatible Materials Testing) OR (Materials Testing, Biocompatible) OR (Biocompatibility Testing) OR (Testing, Biocompatibility) OR (Testings, Biocompatibility) OR (Hemocompatibility Testing) OR (Hemocompatibility Testings) OR (Testing, Hemocompatibility) OR (Testing, Hemocompatible Materials) OR (Hemocompatible Materials Testing) OR (Materials Testing, Hemocompatible) OR (Physiologic Calcification) OR (Calcification, Physiological) OR (Physiological Calcification) OR (Bone Mineralization) OR (Mineralization, Bone) OR (Mineralization) OR (Mineralisable) OR (Mineralized) OR (Bone Formation) OR (Ossification) OR (Ossifications) OR (Osteoclastogenesis) OR (Osteoclastogeneses) OR (Endochondral Ossification) OR (Endochondral Ossifications) OR (Ossification, Endochondral) OR (Ossifications, Endochondral) OR (Physiologic Ossification) OR (Ossification, Physiological) OR (Physiological Ossification) OR (Ossification, Physiologic) OR (angiogeneic) OR (angiogenically) OR (angiogenicity) OR (angiogenics) OR (angiogens) OR (Proliferation, Cell) OR (Cellular Proliferation) OR (Proliferation, Cellular) OR (Cell Multiplication) OR (Multiplication, Cell) OR (Cell Growth in Number) OR (Cell Number Growth) OR (Growth, Cell Number) OR (Number Growth, Cell) OR (gene expression) OR (odontogenic) OR (odontogenous) OR (osteogene) OR (osteogeneic)
